# Role of long noncoding RNA in regulating HIV infection—a comprehensive review

**DOI:** 10.1128/mbio.01925-23

**Published:** 2024-01-05

**Authors:** Noa Amir, Ran Taube

**Affiliations:** 1 The Shraga Segal Department of Microbiology Immunology and Genetics, Faculty of Health Sciences, Ben-Gurion University of the Negev, Negev, Israel; Albert Einstein College of Medicine, Bronx, New York, USA

**Keywords:** human immunodeficiency virus, long noncoding RNA, HIV latency

## Abstract

A complete cure against human immunodeficiency virus (HIV) infection remains out of reach, as the virus persists in stable cell reservoirs that are resistant to antiretroviral therapy. The key to eliminating these reservoirs lies in deciphering the processes that govern viral gene expression and latency. However, while we comprehensively understand how host proteins influence HIV gene expression and viral latency, the emerging role of long noncoding RNAs (lncRNAs) in the context of T cell activation, HIV gene expression, and viral latency remain unexplored. This review dives into the evolving significance of lncRNAs and their impact on HIV gene expression and viral latency. We provide an overview of the current knowledge regarding how lncRNAs regulate HIV gene expression, categorizing them as either activators or inhibitors of viral gene expression and infectivity. Furthermore, we offer insights into the potential therapeutic applications of lncRNAs in combatting HIV. A deeper understanding of how lncRNAs modulate HIV gene transcription holds promise for developing novel RNA-based therapies to complement existing treatment strategies to eradicate HIV reservoirs.

## INTRODUCTION

### Human immunodeficiency virus (HIV)

Antiretroviral therapy (ART) has effectively restricted the transmission of HIV and improved clinical outcomes of viral carriers. Nonetheless, eliminating the HIV reservoirs and achieving a total cure for HIV infection remains challenging. This is due to the presence of a transcriptionally dormant yet replication-competent provirus, which is integrated in the host’s genome. These proviruses endure within long-lasting cellular reservoirs, primarily consisting of memory-resting CD4+ T cells and various cells of the myeloid lineage (1, 2). These reservoirs exhibit remarkable stability and resilience, rendering them resistant to the impacts of both ART and the immune defenses. Consequently, they represent a substantial barrier to completely eliminating viral infection. Furthermore, in the majority of individuals living with HIV, discontinuing ART results in reoccurrence of viral loads, typically occurring within weeks following the cessation of treatment (3–6). As T cell stimulation triggers activation of proviral transcription, one strategy that has been proposed to eliminate the HIV reservoirs is a “shock and kill” approach, which utilizes latency-reversing agents (LRAs) to first specifically activate dormant HIV-infected T cells and facilitate cell death by viral cytopathic effects or immune-mediated killing. This step is done in the presence of ART; thus, no additional rounds of HIV replication occur (7–9). On the other hand, there is a concept known as the “block and lock” approach, which aims to liberate HIV-infected individuals from the need for continuous ART by inhibiting HIV transcription and inducing a profound state of latency. Despite initial promise, these strategies, along with various other attempts, have unfortunately fallen short of achieving substantial clinical effectiveness. This underscores the pressing requirement for alternative therapies with the potential to eradicate the viral reservoir (10–15).

To successfully eliminate the HIV reservoirs, a detailed understanding of the molecular events that lead to its establishment and maintenance is required. These include epigenetic constraints that suppress proviral gene transcription and are important for establishment of HIV latency (16, 17). Furthermore, within the infected T cells, minimal levels of basal and elongating transcription factors, coupled with the absence of the viral trans-activator of transcription (TAT), collectively contribute to keeping proviral transcription levels well below detectable thresholds (18, 19). In both the virus and cells, transcription initiation commences with the recruitment of RNA polymerase II (RNAPII) by general transcription factors and the mediator to the transcription starting site (TSS) at promoters, thereby forming a pre-initiation complex. Subsequently, RNAPII clearance from the promoter is facilitated by the phosphorylation of its carboxy-terminal domain (CTD) at serine 5 by TFIIH/CDK7. However, in the majority of genes, RNAPII halts its transcriptional activity at approximately 25–60 nucleotides away from the TSS, awaiting specific signals to resume productive elongation. This phenomenon, known as promoter-proximal pausing, is followed by the release of RNAPII and represents a pivotal rate-limiting step of transcription. These steps are intricately regulated by negative and positive transcription factors (20, 21). These include pausing-inducing factors comprised of the DRB sensitivity-inducing factor (DSIF), the negative elongation factor (NELF) on one side, and positive transcription elongation factor b (P-TEFb) and super elongation complex (SEC) on the other side. P-TEFb and SEC collaborate synergistically to promote the release of paused RNAPII and to facilitate transcription elongation. Within this partnership, CDK9, a component of P-TEFb, phosphorylates key players such as SPT5, NELF, and the CTD of RNAPII. Simultaneously, the catalytic subunit of SEC, ELL2, plays a crucial role by preventing RNAPII from backtracking and, as a result, enhances the processivity of transcription (22–24). In the context of HIV, the viral TAT protein enhances transcriptional elongation by binding to the nascent TAR viral RNA and recruits P-TEFb and SEC to the viral promoter (25–27). Nevertheless, despite extensive studies on the transcriptional control of metazoan and their contribution to the understanding of the mechanisms that regulate HIV transcription, our comprehension of how HIV latency is established and sustained remains incomplete. Furthermore, the specific host factors responsible for controlling this critical step have yet to be fully identified (28).

### Noncoding RNA (ncRNA) and their mechanisms of function

**Fig 1:**
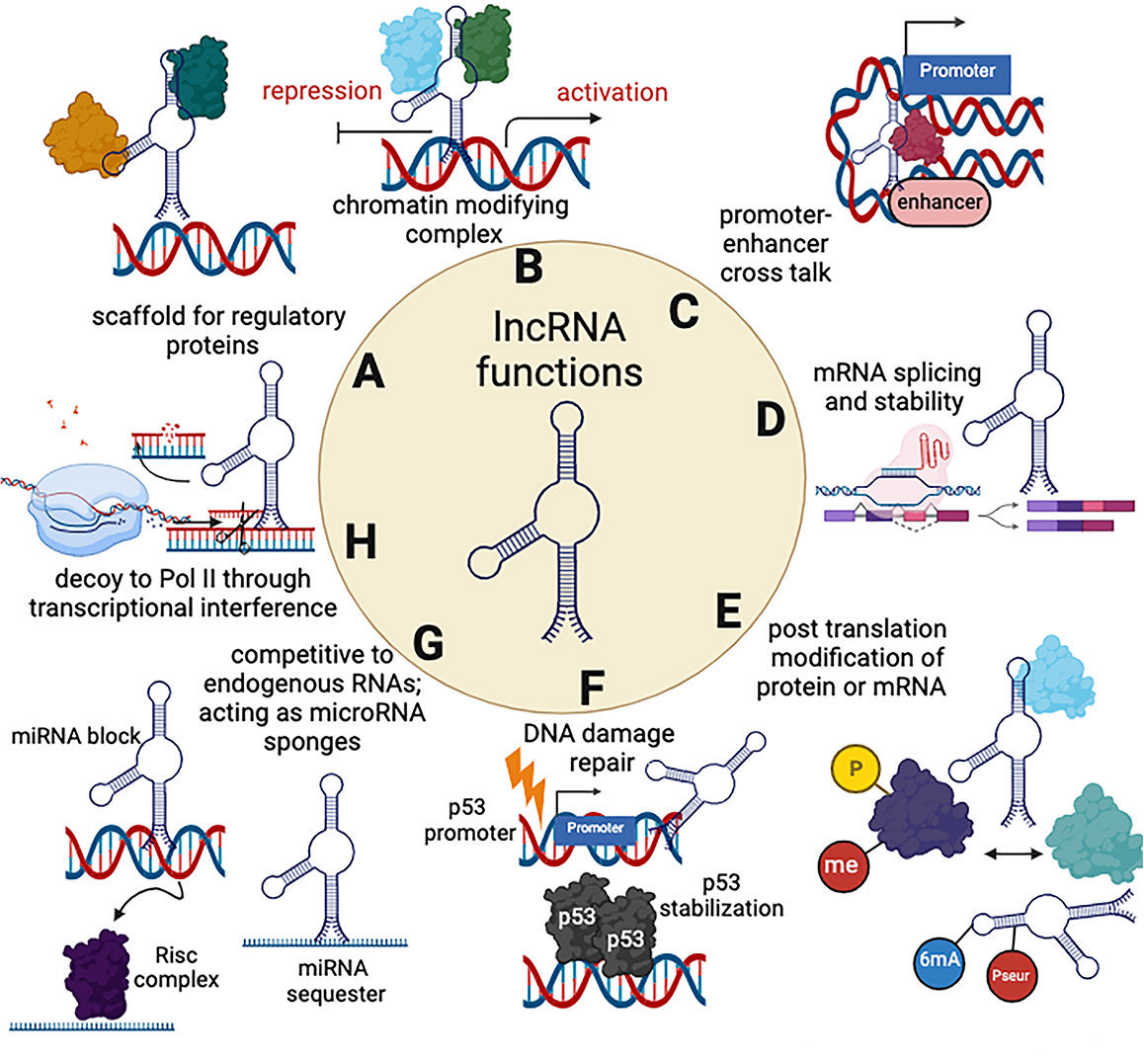
Mechanisms for lncRNAs functions. Noncoding RNAs exhibit diverse regulatory functions. (**A**) lncRNAs can act as a scaffold for protein assembly and mediate gene regulation. (**B**) lncRNAs can recruit epigenetic chromatin modifiers to specific gene promoters, thereby mediating activation or repression of cellular or viral gene expression. (**C**) lncRNAs can form loops between promoter and enhancer regions within genes and mediate enhancer functions. (**D**) lncRNAs can bind to splicing factors or the boundary site between the intron and exon of pre-mRNA to affect RNA splicing. (**E**) lncRNAs can induce protein or RNA post-transcriptional modifications. (**F**) lncRNA can mediate DNA damage repair and affect apoptosis through stabilizing p53. (**G**) lncRNAs compete with miRNAs to bind mRNAs, thereby blocking miRNA-induced silencing through the RNA-induced silencing complex (RISC), thus increasing mRNA translation. (**H**) lncRNAs can serve as miRNA or protein decoys to sequester miRNAs from their mRNA targets.

In recent years, there has been a dramatic paradigm shift in our understanding of the human transcriptome and the fact that it consists not only of protein-coding genes but also of noncoding RNAs that do not possess any protein-coding potential. These noncoding transcripts vary widely, spanning from 150 nucleotides (nt) to as much as 100 kilobases (kb). Those exceeding 200 nt are categorized as long noncoding RNAs (lncRNAs). Most lncRNAs are transcribed by RNAPII, possess a 5′-end cap, undergo splicing, and receive a polyadenylated tail at their 3′-end (29). lncRNAs can be categorized based on their functions into two main groups: cis-acting and trans-acting lncRNAs. Cis-acting lncRNAs operate close to the coding genes that they regulate, typically within a range of up to 10,000 nucleoatides, either upstream or downstream of their target gene. In contrast, trans-acting lncRNAs function from a distance and are typically identified based on their expression levels. Furthermore, lncRNAs can be classified by their position relative to protein-coding genes, leading to categories such as intergenic (located between genes), intronic (found within introns of genes), overlapping (overlapping with coding or noncoding transcripts), sense (transcribed from the same strand as a gene), and antisense (transcribed from the opposite strand of a gene). Finally, ncRNAs can be categorized by their specific functions. This includes ncRNA-activating molecules, microRNA (miRNA) primary transcripts, piwi-interacting RNA primary transcripts, and competing endogenous RNAs, each of which plays distinct roles in cellular processes and gene regulation (30, 31). lncRNA expression is cell type specific and they exhibit distinct subcellular compartments, including subcellular organelles within the nucleus and cytoplasm (32). Their expression also varies between cell development and differentiation stages, as they play a key role in controlling these steps. Other processes where lncRNAs play a significant role include gene expression regulation, cell cycle control, DNA damage response, immune response, stem cell pluripotency, RNA processing (splicing), translation, and post-translation modification processes (reviewed in references 33–37). Accumulated reports also indicate that lncRNAs regulate antiviral immune responses via transcription of key immune modulators like interferon type I (31). The mechanisms by which lncRNAs exert their effects are highly diverse and involve several pathways (Fig. 1). One such mechanism affects the chromatin landscape. This can be achieved by either binding directly to chromatin regulatory proteins, or by recruiting chromatin remodeling factors to specific genomic loci. This interaction can (i) alter the chromatin’s epigenetic landscape, leading to changes in gene expression (38); (ii) regulate splicing and stability of messenger RNAs (mRNAs); (iii) act as decoys (39–41); or (iv) serve as a scaffold for other transcription factors. Indirect effects of lncRNAs on transcriptional and post-transcriptional gene expression are also possible, mainly via interacting with other microRNAs and proteins (42, 43). They can also function as enhancers to facilitate promoter–enhancer interactions (44, 45), act as decoys of RNAPII for transcriptional interference and facilitating mRNA splicing (46), and modify target protein expression and functions by changing their phosphorylation states (47). Overall, it is not surprising that lncRNA transcription is often perturbed in many human diseases, including cancer and neurodegenerative disorders (48, 49).

One of the fundamental mechanisms through which lncRNAs exert their effects is by interacting with miRNAs. Through these interactions, lncRNAs play a regulatory role by modulating the levels of miRNAs. They can also act as molecular sponges, competing with miRNAs for specific binding sites in the noncoding regions of mRNAs. By doing so, lncRNAs effectively counteract the transcriptional repression that miRNAs would otherwise impose on these mRNA genes, thus fine tuning gene expression and regulation (50, 51). The fact that some lncRNAs can also be processed into miRNAs highlights the strong interactions among these two subclasses of ncRNAs (52, 53). lncRNAs have been shown to respond to DNA damage and to mediate DNA repair. For example, estrogen-regulated lncRNA *CUPIDI/2* promotes breast cancer by inhibiting cell cycle arrest upon DNA damage (54). lncRNA *LINK-A*, lncRNA-*HIFCAR* and lncRNA*-LET*, are also good examples of an overall effect on tumor growth (55, 56).

#### Synopsis

To develop effective therapeutic approaches for eradicating the latent HIV reservoirs, it is essential to gain a comprehensive understanding of how HIV gene expression and latency are intricately regulated (36, 57–59). While we have made significant progress in deciphering how host proteins govern HIV gene expression and viral latency, there remains a crucial knowledge gap concerning the emerging role of ncRNAs, specifically lncRNAs, in the context of T cell activation, HIV gene expression, and viral latency. This review aims to consolidate our current knowledge regarding the impact of lncRNAs on the regulation of HIV infection. The effects of lncRNAs on HIV gene expression can be either positive or negative, and understanding their specific modes of action holds promise for developing therapeutic strategies. Selected ncRNAs may be attractive targets for treating viral infection and latency (60–63).

### lncRNAs that activate HIV gene expression and infection

#### Metastasis-associated lung adenocarcinoma transcript 1 (*MALAT1*)

*MALAT1* was initially discovered as a prognostic marker in non-small cell lung cancer (NSCLC). This lncRNA has been linked to the progression and prognosis of NSCLC, making it a subject of interest in cancer research and potentially serving as a valuable marker for assessing the disease’s clinical outcomes (64, 65). In the context of HIV, *MALAT1* promotes HIV-1 gene expression by modifying the epigenetic landscape around the viral promoter. It inhibits the recruitment of the catalytic subunit EZH2 of the polycomb repressive complex 2 (PRC2), thus preventing the methylation of histones around the viral promoter, and ultimately leading to elevated HIV-1 gene expression (66, 67). Moreover, *MALAT1* expression levels are also increased upon treatment of infected cells with latency reversing agents, LRAs, confirming that *MALAT1* can reactivate HIV gene expression and promote latency reversal (67).

#### HIV-1-enhanced lncRNA (*HEAL*)

Through genome-wide expression analysis of lncRNAs in HIV-1-infected primary monocyte-derived macrophages, Tariq Rana’s group identified 1,145 differentially expressed lncRNAs, including *HEAL* (68). *HEAL* is upregulated upon HIV-1 infection of macrophages, microglia, and CD4+ T lymphocytes. In HIV-infected peripheral blood mononuclear cells (PBMCs), *HEAL* also exhibits elevated levels. *HEAL* ncRNA plays a crucial role in enhancing the replication of various HIV-1 strains, and its depletion effectively inhibits infection by X4-tropic, R5-tropic, or dual-tropic HIV strains. Conversely, overexpression of *HEAL* can rescue this inhibition and restore viral infection. The mechanism underlying *HEAL’s* action involves its association with the RNA-binding protein FUS. This complex binds to the HIV promoter and enhances the recruitment of the p300 histone acetyltransferase, which activates HIV transcription by inducing histone H3K27 acetylation and enriching P-TEFb on the HIV promoter. Additionally, the *HEAL*-FUS complex is enriched at the promoter of the cyclin-dependent kinase 2 gene, CDK2, thereby promoting CDK2 expression. Knockdown of *HEAL* prevents HIV-1 reactivation in T cells and microglia when subjected to azidothymidine disruption treatment *in vitro*. In summary, silencing *HEAL* or disrupting the *HEAL*-FUS ribonucleoprotein complex presents a promising epigenetic strategy for eradicating viral reservoirs and working toward a cure for HIV-1/AIDS (68).

#### uc002yug.2

Huan et al. investigated the role of the lncRNA *uc002yug*.*2*, also named *LINC-01426*, in HIV-1 replication and reactivation (69). This lncRNA is 2,564 bp in length, and is derived from the *linc01426* gene that is located on chromosome 21. *uc002yug.2* nRNA was initially identified to be over-expressed in esophageal squamous cell carcinoma. In the context of HIV, *uc002yug.2* plays a crucial role in viral replication and latency. It can enhance HIV-1 replication by activating the viral LTR promoter in both cell lines and primary CD4+ T cells. Mechanistically, the expression of *uc002yug.2* leads to the induction of HIV-1 replication in a dose-dependent manner. This is achieved by its role in facilitating the alternative splicing of the Runt-related transcription factor 1 (RUNX1) pre-mRNA. Specifically, *uc002yug.2* promotes the production of an isoform known as RUNX1a while concurrently reducing the levels of other isoforms, namely, RUNX1b and RUNX1c. This alteration in the balance of RUNX1 isoforms contributes to the enhancement of HIV-1 replication (69). Further investigation confirmed that RUNX1b and RUNX1c, but not RUNX1a, strongly inhibit HIV-1 replication particularly when combined with CBF-β (70). Simultaneously, depletion of uc002yug.2 reduces levels of RUNX1a and increases levels of its RUNX1b/c isoforms, overall inhibiting HIV replication and infectivity. Stimulation of ACH-2 T cells that stably express uc002yug.2 with phorbol 12-myristate 13-acetate (PMA) moderately enhances the expression of HIV-1 compared to cells stably expressing *uc002yug*.2 alone. However, most of the latent HIV-1 can be activated by uc002yug.2, and in combination with suberoylanilide hydroxamic acid (SAHA), it further synergizes HIV gene expression. In Jurkat T cells, *uc002yug.2* does not always down-regulate RUNX1 subtypes, indicating that a different mechanism is employed by *uc002yug*.*2* ncRNA on activation of HIV-1 replication. In another mode of action, *uc002yug.2* lncRNA promotes the expression of HIV TAT, implying that TAT plays a key role in *uc002yug.2*-mediated enhancement of HIV replication and activation of latent HIV-1 (69). Additional research will be required to understand the effects of *uc002yug.2* lncRNA on HIV-1 regulation of infection. It may be that other putative isoforms of this lncRNA play ait role in modulating the predominant isoform in cell lines or in the case of primary cells (70–72).

### lncRNAs that prevent p53-mediated apoptosis in HIV-infected cells or induce senescence

The study conducted by Barichievy and colleagues provides valuable insights into the interplay between HIV-1 infection, DNA damage response, and cell survival. According to their findings, HIV infection of macrophages induces double-strand breaks (DSBs) in the cellular DNA. Furthermore, this DNA damage triggers the activation of the tumor suppressor protein p53. In many cases, when p53 is activated due to severe DNA damage, it can lead to cell cycle arrest, DNA repair, or apoptosis to eliminate cells with irreparable DNA damage, thereby preventing the propagation of genetic mutations. However, in the context of HIV-1-infected macrophages, despite the activation of p53, cytopathic effects and apoptosis do not take place. As a result, the infected cells can survive for an extended period. This unique response suggests that HIV-1 has devolved mechanisms to manipulate the cellular DNA damage response to its advantage, allowing infected cells to persist rather than undergo apoptosis. Understanding these mechanisms may have implications for developing therapeutic strategies to target HIV reservoirs and potentially eradicate the virus (73). In response to p53-mediated transcription, two lncRNAs are produced, *lincRNA-p21* and the p21-related ncRNA damage activation (*PANDA*). These transcripts are derived from the CDK1A (asp21) promoter as a response to DSB. *lincRNA-p21* is de-regulated upon HIV infection, leading to its nuclear association with HuR and degradation of the RNA transcript. Simultaneously, the interactions between *lincRNA-p21* and the heterogeneous ribonucleoprotein K (hnRNP-K) are also de-regulated. *lincRNA-21* association with hnRNP-K also promotes apoptosis by localizing hnRNP-K to p53-repressed genes and, in turn, enhances the expression of pro-apoptotic genes. The hnRNP-K-*lincRNA-21* complex also triggers apoptosis by repressing the expression of pro-survival p53 target genes (74). In HIV-infected cells where apoptosis is not initiated, the HuR-*lincRNA-21* complex plays a critical role in sequestering hnRNP-K in the cytoplasm. This event prevents the formation of the pro-apoptotic complex and increases the levels of genes associated with cell survival. One of the target genes regulated by the *lincRNA-21*/hnRNP-K complex, which promotes cell survival, is mitogen-activated protein kinase 1 (MAP2K1). MAP2K1 encodes a protein that phosphorylates extracellular signal-regulated kinase 2 (ERK2), a crucial player in cellular survival pathways. Phosphorylated ERK2 helps maintain cellular survival by participating in various signaling cascades that support cell growth and proliferation.

In infected cells, HIV-1 ensures activated ERK2 phosphorylation expression, thereby leading to cytoplasmic accumulation of hnRNP-K and phosphorylation of the ubiquitin E3 ligase, HDM2 (75). This ensures ubiquitin-mediated degradation of nuclear hnRNP-K and p53, thereby disrupting apoptosis. Appropriate nuclear interactions between *lincRNA-p21* and hnRNP-K can be restored through the inhibition of MAP2K1 or ERK2 or via the combined action of NUTLIN3a and enhanced expression of *lincRNA-21*, demonstrating how control of *lincRNA-21* activity can be critical to apoptosis evasion by HIV-1 in macrophages. Finally, *PANDA*, also a p53-dependent lncRNA, is downregulated upon HIV infection and inhibits apoptosis of infected cells through binding to NF-YA and preventing its recruitment to pro-apoptotic target genes (74, 76). *PANDA* expression levels are also regulated via the p53 pathway, and upon HIV infection, its expression is downregulated. In Jurkat cells, silencing of *PANDA* expression supports p53-mediated apoptosis, while its overexpression induces opposite effects and prevents apoptosis (77). The interplay of the two mentioned lncRNAs may explain the resistance of HIV-infected macrophages to DNA damage due to HIV integration and the low susceptibility of infected cells to cytotoxic effects (78).

#### FAS antisense 1 (*FAS-AS1*)

As lncRNAs are cell specific, different HIV target cells express different lncRNAs and affect HIV gene expression and infection. Tissue-resident macrophages are long-lived myeloid cells that self-renew and, like resting CD4+ T cells, also reside within the HIV-infected reservoirs. Macrophages within the brain or at tissues like microglia, liver Kupffer cells, and alveolar macrophages are infected by HIV and maintain prolonged low levels of viremia at different stages of the viral infection (79). However, unlike CD4+ T cells, macrophages are resistant to virus-induced cell death and apoptosis (80). Moreover, high viral burden, rapid rate of disease progression, and aberrant immune activation in the CD4+ T cell-depleted environment, combined with extending cell survival and ability to sustain viral replication, all contribute to the persistence of HIV in macrophages even in the presence of therapy (78). As such, cell-resident macrophages are a major obstacle in the efforts to eliminate the HIV reservoir. Understanding the mechanisms by which HIV persists in these cells is, therefore, critical. Moreover, the role of macrophage-specific lncRNAs in mediating the long-term survival of HIV is also yet to be explored. One lncRNA reported to be upregulated in macrophages upon HIV infection is *FAS* antisense 1 (*FAS-*AS1 *or SAF*). *SAF* is a 1.5-kb antisense lncRNA transcribed from the intron 1 region of the *Fas* (cell surface death receptor) gene (81). Its expression prevents apoptosis by inducing alternative splicing of the Fas gene and increasing the production of a soluble form (82). Silencing *FAS-AS1* promotes the expression of caspase-3/7 and, therefore, increases apoptosis, which is mediated through FAS. Overall, *FAS-AS1* prevents apoptosis in macrophages infected with HIV-1 (83). Interestingly, *FAS-AS1* does not appear to have these effects in T cells (84).

#### *Tug1* lncRNA

**Fig 2:**
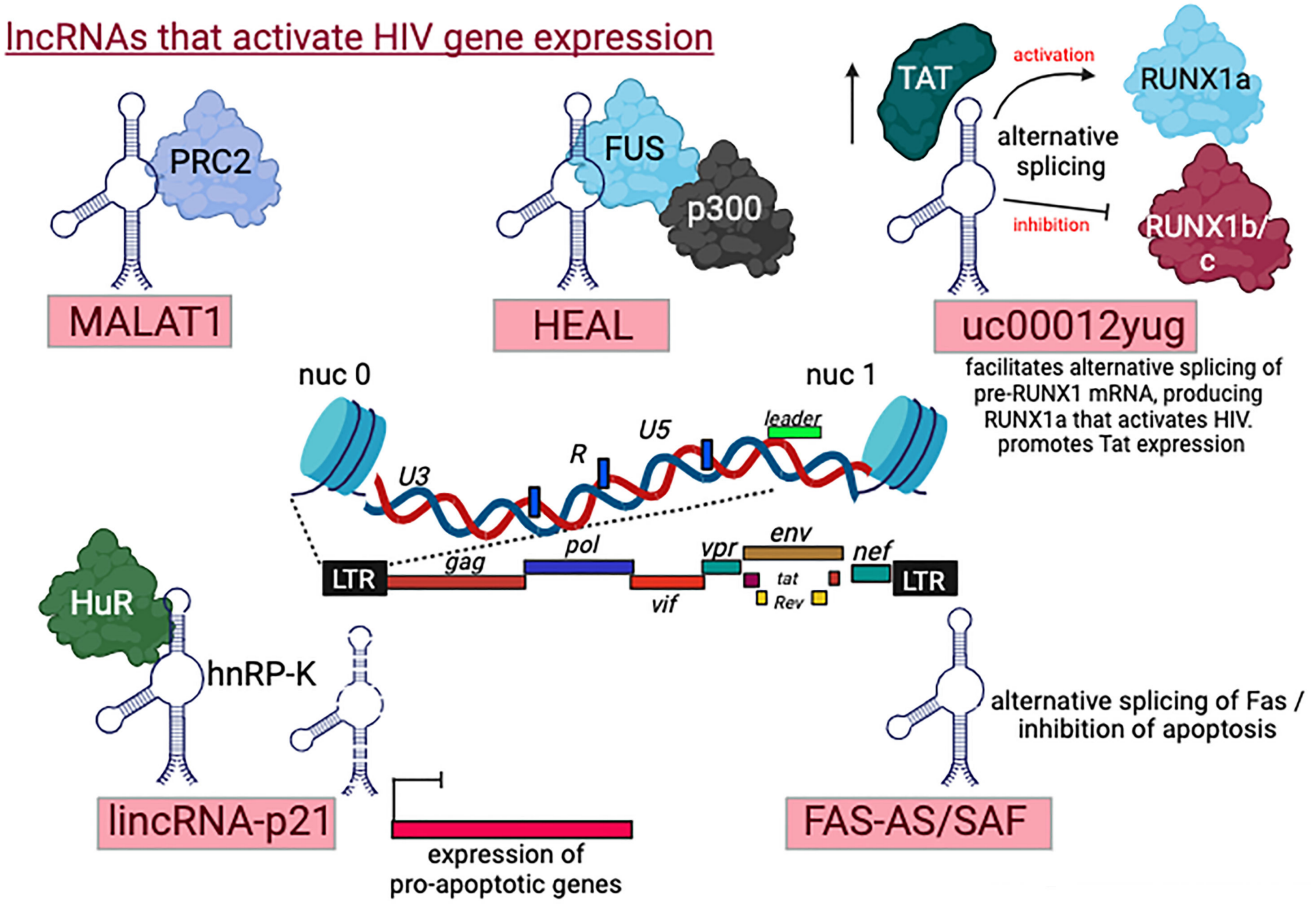
lncRNAs that activate HIV gene expression. A defined class of ncRNAs induce HIV infection by several molecular mechanisms: *MALAT1* promotes HIV-1 gene expression by modifying the epigenetic landscape around the viral promoter, where it suppresses the recruitment of the PRC2/EZH2 and thus represses H3K27me3 on the viral promoter; *HEAL* associates with the RNA-binding protein FUS to facilitates recruitment of the p300 histone acetyltransferase, which activates HIV transcription through H3K27Ac and P-TEFb enrichment; *uc002yu**g* plays a crucial role in viral replication and latency and post-transcriptionally enhances HIV-1 replication through activating the viral LTR by facilitating alternative splicing of the Runt-related transcription factor 1 (RUNX1), producing a shorter isoform RUNX1a and decreasing expression of RUNX1b/c isoforms, leading to inhibition of Runx-mediated repression of HIV infection. *FAS-As/SAF* prevents apoptosis in macrophages infected with HIV-1; lncRNA*-p21* depletion during infection inhibits p53-induced expression of pro-apoptotic genes. See text for more details.

With the increased life expectancy in people living with HIV-1, there is still an elevated prevalence of HIV-1-associated neurocognitive disorders as these patients live longer. Several studies have shown that in HIV-infected carriers, senescent cells are accumulated within the aging brain (85), primarily in cells that are target for HIV infection like in mesenchymal stem cells (86), corneal endothelial cells (10), CD8+ T cells (87), CD4+ T cells (88), microglia (89), and astrocytes (90). In a study that aimed to explore the effects of soluble TAT on the senescence of astrocytes, the authors report the role of *Tug1* lncRNA as a player in inducing senescence in the presence of TAT. Soluble TAT is known to be secreted from HIV-infected cells like astrocytes, microglia, and macrophages and crosses the blood-brain barrier into the CNS, where it exerts its cytotoxicity directly on glial and neuronal cell leading to the release of neuroinflammation soluble factors, along with oxidative stress and neuronal damage. In their study, Pillai et al. show that TAT increases SA-β-gal activity, p21 and p16 expression, and induces cell cycle arrest, reactive oxygen species (ROS) expression , the formation of senescence-associated heterochromatin foci, and the expression of proinflammatory cytokines. Notably, Tug1 lncRNA expression levels in astrocytes were also elevated. *Tug1* lncRNA promotes cell proliferation and is a direct target of p53 with a role in repressing specific genes involved in cell cycle regulation (91). Silencing of *Tug1* lncRNA inhibits most of the above mentioned effects of TAT. Their results link *Tug1* to astrocyte senescence that is induced by TAT. These results elucidate the understanding of the role of *Tug1* in driving cellular senescence in astrocytes and accelerating aging processes. Overall, *Tug1* lncRNA could be positioned as a therapeutic target to control accelerated or premature aging in HIV carriers (92) (Fig. 2).

### lncRNAs that suppress HIV infection

#### NRON

The research conducted by Imam et al. involved the screening of over 90 lncRNAs that are known to be associated with various diseases, including multiple types of cancers. This screening was performed in two stimulated T cell lines infected with HIV: Jurkat T and J1.1 cells. They aimed to identify lncRNAs that exhibited consistent changes in expression levels in both latency cell models upon HIV infection. One specific lncRNA that captured their attention was the noncoding repressor of the nuclear factor of activated T cells (NFAT), known as *NRON*. *NRON* lncRNA expression was consistently downregulated in both the Jurkat T cell line and J1.1 cells following HIV infection. *NRON* is known for its role in regulating the activity of the transcription factor NFAT, which plays a critical role in T cell activation. The desregulation of *NRON* may have implications for the immune response and the interplay between HIV and host cells. By identifying *NRON* as a consistently downregulated lncRNA in HIV-infected T cells, this research provided valuable insights into the molecular mechanisms involved in HIV pathogenesis and its impact on T cell function. Understanding the role of *NRON* in the context of HIV infection may open avenues for further research and potential therapeutic interventions (93). *NRON* is approximately 2.7 kb in length, composed of three exons and acts as a repressor of NFAT, which enhances HIV-1 gene expression in primary CD4+ T cells. In resting T cells, NFAT is phosphorylated and is sequesters in the cytoplasm by *NRON* lncRNA . Upon knockdown of *NRON*, HIV-1 replication is enhanced relatively to control Jurkat T cells. In these conditions, NFAT activity is elevated, subsequently activating the HIV promoter. Mechanistically, *NRON* binds to NFAT and inhibits its nuclear translocation. Interestingly, during HIV infection, Nef can decrease *NRON* levels, while Vpu increases Nef levels. Thus, Nef and Vpu join to regulate NFAT activity by altering *NRON* expression levels. In Vpu-expressing cells, NFAT transcription activity is reduced, while in Nef-expressing cells, NFAT activity is increased. Upon HIV-1 infection of CD4+ T cells, the levels of *NRON* are decreased, leading to the elevation of NFAT expression in the nucleus and activation of the HIV-1 LTR. This process, coupled with T cell activation by NFAT, ultimately enhances HIV-1 replication. Subsequently, in the viral life cycle, Vpu expression results in elevated levels of *NRON*, potentially decreasing T cell activation, NFAT-mediated activation, and cell apoptosis (93). In another study, Li et al*.* showed that *NRON* also contributes to establishing HIV latency by specifically inducing the degradation of TAT. *NRON* directly links TAT to the ubiquitin/proteasome components, including CUL4B and PSMD11, thus facilitating TAT degradation. Specifically, depletion of *NRON*, in combination with treating cells with histone deacetylase inhibitor, significantly reactivates HIV production from the HIV-1 latently infected primary CD4+ T lymphocytes (94).

#### Nuclear paraspeckle assembly transcript 1 (*NEAT1*)

*NEAT1* is transcribed from a specific locus in human chromosome 11 by RNAPII to produce two distinct isoforms, *NEAT1_1* (3.7 kb) or *NEAT1_2* (22.7 kb), as a result of alternative 3′-end processing. *NEAT1_1* is polyadenylated, whereas *NEAT1_2* lacks the poly(A) tail and instead contains a triple helix structure at its 3′ terminus; therefore, it is processed differently. *NEAT1* has been recognized to localize into paraspeckles in the nucleus. However, only *NEAT1_1*, but not *NEAT1_2*, is required for paraspeckle formation (95). Within the paraspeckles, *NEAT1* plays critical roles in multiple cellular and pathophysiology conditions, such as innate immune effects, organ development, cancer, and neurodegenerative diseases. Upon HIV infection, the expression levels of *NEAT1* are elevated, and HIV transcripts are co-localized to nuclear paraspeckle. Knocking down *NEAT1* results in reduced numbers of paraspeckles, confirming that *NEAT1* acts as a scaffold for nuclear paraspeckles formation (96). This report also shows that the knockdown of *NEAT1* induces HIV production by increasing nucleus-to-cytoplasm export of Rev-dependent instability element (INS)-containing HIV-1 mRNAs. For cellular transcripts, in the case of stress and the requirement for rapid RNA synthesis, paraspeckles serve as retention organelles for storing unspliced mRNA, some of which may be subjected to RNA-processing splicing events. This sorted RNA may also be a way to stabilize and maintain RNAs that might otherwise be degraded, thereby offering a more rapid and resource-efficient way than *de novo* RNA biosynthesis. HIV adopted this mechanism to store its excess unspliced INS-containing viral RNAs. Within paraspeckles, *NEAT1* ensures the storage of unspliced HIV transcripts, thereby keeping excess unspliced INS containing viral RNAs (96, 97). Recently, Liu et al. employed CRISPR/Cas9 to knock down *NEAT1* and demonstrated its role in innate immunity against viral infection. Knockdown of *NEAT1* in T cells had no effects on T cell activation but led to increased sensitivity of cells to apoptosis relative to control cells. They confirmed that upon depletion of *NEAT1*, HIV replication was elevated. Moreover, following activation of resting CD4+ T primary cells, *NEAT1* expression was downregulated. These findings imply that HIV-1 infection exploits the normal downregulation of *NEAT1* lncRNAs in activated CD4+ T cells to enhance viral replication (98). Finally, recent studies showed that *NEAT1* expression in plasma was correlated with CD4+ T cell counts, indicating that *NEAT1* may be a promising biomarker for disease progression (99).

#### *7SK* ncRNA

**Fig 3:**
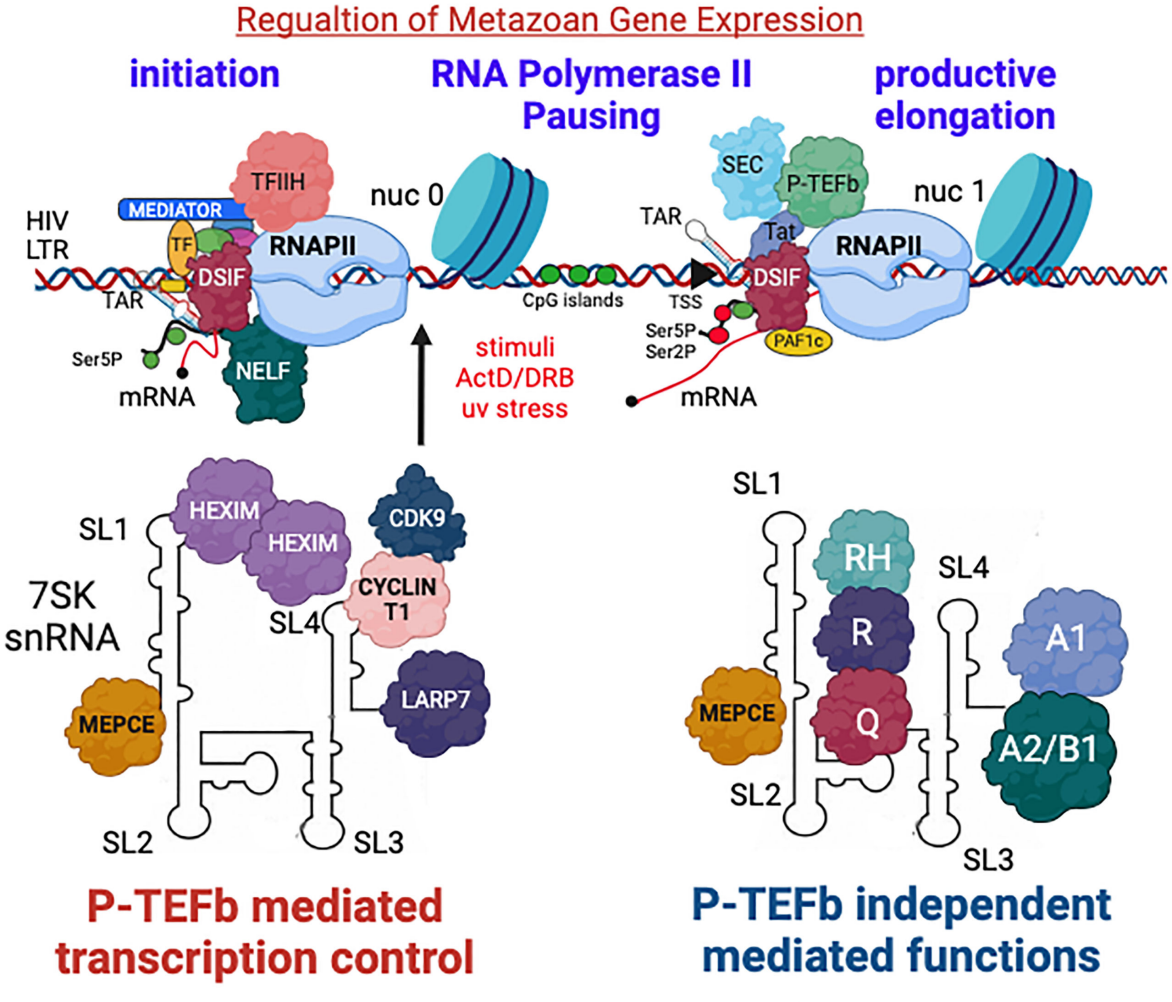
A P-TEFb-7SK snRNP interplay within cells dictates metazoan or HIV gene transcription elongation. In cells, P-TEFb is tightly regulated in active or inactive complexes that are ready for gene expression regulation. To promote RNAPII pause-release and productive transcription elongation, P-TEFb must be released from its inhibitory 7SK snRNP complex. *7SK* ncRNA acts as a scaffold for the assembly of protein partners, including MePCE, LARP7, and HEXIM/2; binds CYCLIN T1; and hijacks P-TEFb from TAT to inactivate CDK9.

Over the years, HIV has laid the foundations for the current understanding of metazoan gene expression regulation. Similar to cellular genes, the integrated HIV genome is transcribed by RNAPII and initiates transcription from TSSs. However, RNAPII quickly pauses and remains stably engaged with a short nascent RNA, without capability to elongate the mRNA. To induce RNAPII release and productive elongation, external signals facilitate the recruitment of the positive transcription elongation factor b, P-TEFb, to gene promoters. P-TEFb phosphorylates the CTD of RNAPII on Ser2, SPT5, and the RD subunit of NELF to mediate productive elongation of transcription (26, 33, 100, 101). As a central hub for transcription in cells, P-TEFb exists in an equilibrium of an active free complex and an inactive form that is tightly associated with the 7SK small nuclear ribonucleoprotein (snRNP) (24, 102, 103). *7SK* ncRNA is an abundant and conserved 330–332 nt RNA transcribed by RNAPII (104). Significant advances in its structural biology have improved our understanding of *7SK* RNA biogenesis, protein assembly, and biological functions. Several secondary structure models of the RNA have been proposed, revealing that the ncRNA consists of four stem-loop domains (SL1–4) with intervening single-stranded RNA linkers (104, 105). In 7SK snRNP, the *7SK* ncRNA acts as a scaffold for assembling its protein partnersAt its 5′end. *7SK* ncRNA binds to the methylphosphate capping enzyme (MePCE), which adds to the 5′-end of the RNA a monomethyl cap onto the γ phosphate (106). On its 3′ U-rich motif end, *7SK* ncRNA is protected and stabilized by the LA protein. LA protein detaches from the 7SK complex and is replaced by the La-related protein 7, a lupus antigen (La) module LARP7, thereby becoming a member of the *7SK* snRNP through its La and RRM1 motifs (107, 108). Both LARP7 and MePCE interact with each other in the absence of the 7SK RNA. However, their association is strengthened upon RNA assembly, as they protect *7SK* ncRNA from end degradation (109). Depletion of MePCE or LARP7 destabilizes 7SK snRNP, leading to the release of P-TEFb for productive transcription elongation (108, 110–114). Thus, it is not surprising that mutations in MePCE or LARP7 are seen in numerous human malignancies, including gastric, breast, and cervical cancers. Another protein partner of *7SK* snRNA includes the hexamethylene bisacetamide (HMBA)-induced mRNA, HEXIM/2 proteins (115). Via their KHRR basic motif, HEXIM proteins cooperatively dimerize and bind to the 5′-stem loop GAUC region at the proximal and the distal part of SL1. HEXIM1 then acquires an auto-inhibitory confirmation, exposing its coiled-coil C-terminal region as a platform for CYCLIN T1 binding, and subsequently blocks the ATP binding pocket of the CDK9 kinase (116–122). Phosphorylation of CDK9 at threonine 186 further enhances the incorporation of P-TEFb onto the 7SK inactive complex (123). Finally, through its C-terminal RRM3 motif, LARP7 also interacts with CDK9 to stabilize P-TEFb incorporation onto the complex (109, 124). Under steady-state conditions, most 7SK snRNP is associated with P-TEFb. For active transcription, 7SK snRNP dissociates, allowing P-TEFb to transition into a free active complex. This occurs in response to various stimuli like DNA damage, cardiac hypertrophy, and HIV infection. The phosphorylation events of CDK9, HEXIM1, CYCLIN T1 acetylation, and MePCE cleavage cooperate to induce P-TEFb release from 7SK snRNP (125). Importantly, P-TEFb release occurs in the nucleus near chromatin, thereby facilitating its recruitment to gene promoters or enhancers. However, how P-TEFb is brought onto gene promoters is a question of extensive research. BRD4 is considered a key player in recruiting P-TEFb to chromatin (126, 127). At the human ribosomal gene promoters, the DEAD-box RNA helicase DDX21 interacts with 7SK/P-TEFb and, in cooperation with the WD40 domain-containing protein WRD43, facilitates P-TEFb release and promoter recruitment (128). 7SK snRNP also interacts with the repressive H4R3me2 histone at the enhancers mark and, together with the Jumonji C domain-containing protein JMJD6, enhances the release of RNAPII in a BRD4-dependent manner (129). TRIM28/KAPI, a Kruppel-associated box-associated protein, also has been shown to recruit 7SK-P-TEFb to various promoters through LARP7, enabling P-TEFb-mediated elongation of transcription (130). The SRSF2 splicing factor also assembles into the 7SK complex and binds gene promoters to mediate pause-release (131). In addition, MePCE is also directly associated with histones to target P-TEFb to chromatin. However, the role of the 7SK-P-TEFb complex is unclear in this context (132). Finally, 7SK snRNP interaction with the C2H2-type zing-finger protein CTIP2a repressor enhances the inhibition of P-TEFb (132–134) (Fig. 3).

The other half of 7SK snRNP that is not associated with P-TEFb interacts with a range of RNA-binding proteins that are currently the subject of extensive studies, mainly in the context of neuronal development and spliceosome biogenesis. Among them, *7SK* ncRNA is associated with the RNA helicase A and heterogeneous nuclear ribonucleoproteins A1, A2/B1, R, and Q (121, 135–137). Moreover, *7SK* snRNA modifications like methylation mediated by METTL3 promote transcriptional activity, presumably releasing P-TEFb for transcription. This step is mediated by the estrogen growth factor signaling pathways that induce phosphorylation of METTL3 and transcriptional activation, resulting in methylation of *7SK* ncRNA and release from HEXIM/P-TEFb (138). Other known post-transcriptional modifications of 7SK ncRNA include pseudouridylation (139) and m6A (140). Their functional significance to transcription elongation is under study. In the context of HIV, Tat extracts P-TEFb from its inactive 7SK complex by competing with Hexim1 on the binding to cyclin T1 and hijacking P-TEFb to the viral promoter for pause-release and transcription elongation. Like Tat, BRD4 Rbm7 and Myc act similarly and extract P-TEFb from its 7SK snRNP inactive complex (141–143). UV light, actinomycin D, and P-TEFb inhibitors like HMBA or suberoylanilide hydroxamic acid (SAHA) also release P-TEFb from the 7SK snRNP (144, 145).

#### AK130181

Searching for novel lncRNAs that act as factors that control HIV latency, Li et al. investigated the top 10 genes and lncRNAs that were identified by comparing PBMCs that are infected with HIV to control cells. Gene ontology (GO) analysis of their top 20 lncRNAs revealed that these hits are associated with viral-host interactions, innate response, cytokine receptor interactions, JAK-STAS, and NF-kB signaling pathways. Among the identified lncRNA, *AK130181* and *IFIT3* were highly expressed compared to the control cells that were not infected. *AK130181* is highly expressed in resting CD4+ T cells compared to activated T cells. Depletion of *AK130181* led to activation of the HIV promoter and elevated HIV gene expression in primary CD4+ T cells. Moreover, *AK130181* overexpression inhibited viral replication. Their suggested model depicts that *AK130181* lncRNA suppresses the NF-kB-dependent HIV-1 LTR activity and inhibits HIV replication. Finally, *AK130181* contributes to latency since its silencing promotes the reactivation of HIV in infected primary CD4+ T resting cells isolated from patients (146).

#### Growth arrest-specific transcript 5 (*GAS5*)

**Fig 4:**
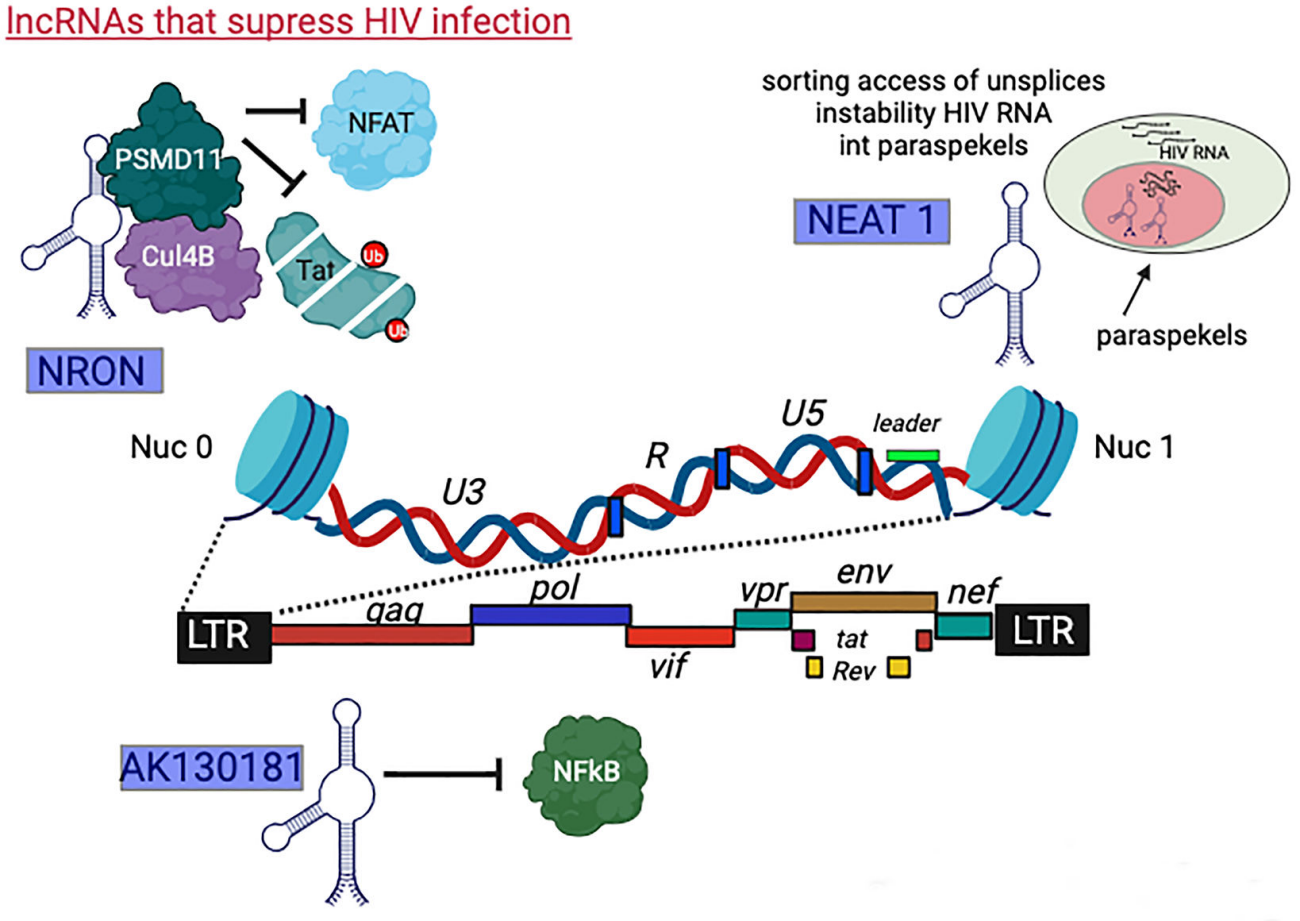
lncRNAs that suppress HIV gene expression. A defined class of ncRNAs inhibit HIV infection by several molecular mechanisms: *NRON* is a repressor of the transcription factor NFAT, which enhances HIV-1 gene expression in primary CD4+ T cells. *NRON* binds to NFAT and inhibits its nuclear translocation; *NEAT* is a vital component of paraspeckles and acts as a scaffold for nuclear paraspeckles formation. Upon viral infection, the expression levels of *NEAT1* are elevated, and HIV transcripts are co-localized to nuclear paraspeckles as unspliced transcripts; *AK130181* is highly expressed in resting CD4+ T cells. It suppresses the NF-kB-dependent activation of the HIV promoter. *AK130181* contributes to latency, since its silencing promotes the activation of HIV in infected primary CD4 T resting cells isolated from patients.

*GAS5* is an inhibitor lncRNA known to prevent tumors’ growth and is often used as a marker for evaluating the effectiveness of chemotherapy and radiation therapy. Studies have shown that it might also inhibit the replication of hepatitis C virus. In the context of HIV, the expression of *GAS5* was first found by Imam et al. to be downregulated during HIV-1 infection (93). Overexpressing *GAS5* leads to reduced levels of HIV infection, while *GAS5* knockdown enhances viral replication. Overexpression of *GAS5* also reduces the levels of HIV-1 *gag* and *pol*, while its knockdown increases overall levels of HIV-1 mRNA. Similar findings were obtained for HIV-p24 and TAT proteins. These reports show that *GAS5* functions via miR-873, which is suppressed by *GAS5*. Indeed, miR-873 increases HIV-1 replication. This study on *GAS5* revealed that it may be a biomarker for the efficacy of antiviral drugs (147). In conclusion, *GAS5* can repress the replication of HIV-1 and potentially acts as a competing endogenous RNA that reduces the activity of the microRNA, miR-873 (148) (Fig. 2, lncRNAs that suppress HIV infection) (Fig. 4).

### Translation and clinical implications of lncRNAs as targets for treating HIV infection

Regarding cancer, lncRNAs have positioned themselves as promising targets for treatment (149), exploiting them as targets for eliminating HIV infection and viral latency lags . One direction where there is great success in using lncRNAs for treating cancer is their use as biomarkers in tissues and fluid samples like blood and for diagnosis of disease progression (150). The expression profiles of specific lncRNAs can be used to follow the effects of therapy and the development of drug-resistant cells in human malignancies (151). Nevertheless, identifying lncRNAs that are specifically unique in these disease conditions limits their development into therapeutic tools. A valid question that remains to be asked is how to select the best lncRNA targets that regulate disease progression and define them as therapeutic targets. One assumption is to choose those lncRNAs with the fewest partners that do not compete with other molecules for the same partners. Depending on their localization, nuclear lncRNAs can be targeted by several tools. Chemically modified anti-sense oligos (ASOs) provide a positive effect targeting lncRNAs, leading to their degradation. ASOs can also be used to disrupt the interactions of the lncRNA with its target mRNA or miRNA. However, these observations also minimize the promising effects of ASO as targeting moieties for lncRNAs (152–154). Limitations can stem from toxicity issues (155) and the choice of the correct immunogen. CRISPR gene editing tools like CRISPR interference or CRISPR activation can also be used for targeting lncRNAs via transcription modulation at their promoter (156, 157). Cytoplasmic lncRNAs can be targeted for degradation by using short hairpin RNA, minor interference RNA (siRNA), and miRNA that can act as mimetics or sponges for lncRNAs. Targeting may also include post-transcriptional inhibition, steric structural interference, or the introduction of synthetic oligos. Here, lncRNA mimetic stability, penetration, off-target effects, and tissue pharmacokinetic properties limit the efficient use of these tools in the clinic. Like in other aspects of RNA-based therapeutics, immunogenicity and the lack of delivery vehicles suitable for targeting the organ or cell type of interest are limiting and, often enough, lead to the termination of clinical trials due to low efficiencies of the lncRNA targeting moiety (158). RNA-based therapy can be delivered as naked nucleic acid or via liposomes or viral-mediated particles. However, one of the biggest problems with delivering RNA molecules is their stability in the blood. Encapsulating lncRNA into nanocarriers, chemical modification can increase chemical stability and minimize the immunological repercussions of the RNA (159). Microinjection (160), protamine condensation (161), RNA patches (162), lipid (163), and polymer-based nanoparticles (164–166) are also tools to deliver the RNA vaccine. Also used are advanced formulation techniques, such as lipid and lipid-based nanoparticle (LNP) encapsulation (167), exosome-encapsulated RNA (168), and cell-penetrating peptides (169). Nevertheless, additional research is required to identify the optimal delivery modalities that can be systemically used for targeting specific HIV-1 cells or tissue sites.

The primary current efforts for pioneering an efficient treatment for HIV infection and disease progression are focused on introducing new drugs into the clinic that aim to reduce viral loads and inhibit viral replication. These can be integrated as a preventive drug in the form of pre-exposure prophylaxis or as a conventional highly active antiretroviral therapy (HAART) treatment for HIV carriers. Additional “shock and kill” or “block and lock” approaches are being tested for therapy, employing latency-reactivating agents or latency inducers. RNA-based therapeutics, specifically lncRNA, offer great potential as front-runners for designing the next-generation therapeutic platform. Such RNA-based technologies are acclaiming popularity based on the successful developments of the mRNA-based vaccine against SARS-CoV-2. Recently reported use of lncRNAs for diagnosis of HIV includes monitoring of plasma levels of lincRNA-*57762303* and lincRNA *165509129*. The presence of *NEAT1* in plasma has been suggested as a biomarker for viral infection (99). Nevertheless, their effectiveness on HIV infection is currently under investigation.

Relative to numerous cancer-related clinical trials on the effects of lncRNA targeting, only a few studies have been initiated to eliminate HIV infection. In 2022, 61 ongoing clinical trials involved lncRNAs but none for HIV-related infection. The fact that most of the reports on the effects of lncRNAs on HIV infection have been done *in vitro* or in cell cultures further imposes difficulties in translating these encouraging findings into clinical settings, which are aiming to incorporate these targets as part of current anti-viral therapy. Furthermore, the observations that lncRNAs share the same key cellular partners as other ncRNA (cellular or viral) may result in severe side effects. Consistent with the idea of targeting a single noncoding RNA, achieving complete suppression or reactivation of the HIV provirus may not be feasible. However, despite these limitations, we believe that targeting HIV latency via regulatory ncRNAs and primarily lncRNAs may be successful in the near future, displaying high specificity and low toxicity effects compared to current protein-based strategies that aim to limit HIV infection. The successful developments obtained for treating human malignancies, combined with the diverse functions of lncRNAs and the availability of novel delivery tools, will certainly open the way for new strategies to be developed to inhibit HIV infection and eliminate the viral reservoir.
